# Turkish Translation and Cultural Adaptation of the Motor Planning Maze Assessment (MPMA)

**DOI:** 10.3390/bs15040492

**Published:** 2025-04-08

**Authors:** Zeynep Ozdemir, Sevda Asqarova, Teresa May-Benson, Aymen Balikci

**Affiliations:** 1 Department of Occupational Therapy, Graduate School of Health Sciences, Uskudar University, Istanbul 34000, Türkiye; zeynep.ozdemir5@st.uskudar.edu.tr; 2 Department of Occupational Therapy, Faculty of Health Sciences, Uskudar University, Istanbul 34000, Türkiye; sevda.asqarova@uskudar.edu.tr; 3 TMB Education, Norristown, PA 19401, USA; tmb@tmbeducation.com; 4 Sense On Ltd., Istanbul 34000, Türkiye

**Keywords:** motor planning, sensory integration, assessment, praxis, developmental coordination disorder

## Abstract

The MPMA is a performance-based evaluation designed to quickly and accurately screen for motor planning challenges in children between 3 and 12 years old. This test is currently unavailable in Turkish. A systematic, multistage translation process is essential for preserving equivalence between a test’s source and target versions. This study aimed to create a culturally adapted Turkish research version of the MPMA for forthcoming normative data collection, reliability, and validity studies. Based on the literature review and recommendations, a seven-step protocol for translation and cross-cultural adaptation was followed. The procedure encompasses forward and backward translations, expert linguistic evaluation, cognitive interviews, and pilot testing. This seven-step process for translating and culturally adapting the MPMA was completed. Despite the necessity for certain modifications in the synthesis and linguistic analysis stage, there were no semantic or conceptual discrepancies in the forward translations. Some discrepancies occurred between the backward translation and the original version; however, this did not influence the standard administration and scoring of the test. The Turkish adaptation of the MPMA will be a valuable tool for clinical practice and research with the Turkish population. Appropriately translated and culturally adapted assessments, such as the MPMA, will influence both practical applications and research opportunities concerning motor planning in Turkey.

## 1. Introduction

Motor skills significantly influence the development of several domains, including social-emotional (Adolph & Hoch, 2019; Capio et al., 2024), cognitive (Adolph & Hoch, 2019; Cameron et al., 2012; Capio et al., 2024; L. Wang & Wang, 2024), and language (Iverson, 2010; Leonard & Hill, 2014) abilities. Additionally, the development of motor skills has a profound impact on various aspects of life, including the execution of daily tasks, participation in educational activities, and social interactions. Proficient motor skills enhance an individual’s ability to navigate the environment efficiently and independently (Adolph & Hoch, 2019; Capio et al., 2024; Haywood & Getchell, 2020). Moreover, motor skills are essential for assessing developmental progress, as they are readily observable and quantifiable indications of development during childhood (Adolph & Robinson, 2015). The predominant approach for analyzing changes in motor capacities entails evaluating the specific ages at which particular skills are acquired. Extensive research has yielded significant insights into the anticipated timing for acquiring fundamental motor skills in typical development (Adolph & Robinson, 2015; Haywood & Getchell, 2020; Piek, 2006). These age-specific benchmarks are essential for discerning typical or atypical developmental trajectories and are extensively employed in clinical and educational contexts to assess motor development (Gabbard, 2021; Payne & Isaacs, 2020; Piek, 2006). While milestone assessments provide a general overview of developmental progress, they often fail to capture the complexity of motor skills, including the capacity to motor plan, sequence, and execute intentional movements, which are essential for daily functioning (Hallemans et al., 2020; Ivey et al., 2014). There has been a growing emphasis on assessing sophisticated motor skills, such as motor planning, in children across many diagnostic groups (Bäckström et al., 2021; Bhoyroo et al., 2019; Lust et al., 2018; Rinehart et al., 2006; S. Wang et al., 2020). Motor planning is a crucial component of daily motor behavior, forming the foundation of the action repertoire of healthy individuals (Krajenbrink et al., 2020). In addition, motor planning skills play a critical role in children’s ability to perform many activities in their daily lives, such as dressing, playing, or social interaction (Ayres, 2005; Bundy & Lane, 2020; Schaaf & Mailloux, 2015).

Occupational therapists utilizing the sensory integration framework focus on assessing and addressing deficits in motor performance, particularly challenges with motor planning (Ivey et al., 2014). Motor planning skills are considered a facet of the broader process of praxis, a sensory integration function (Bundy & Lane, 2020; Ivey et al., 2014). Praxis is the neural mechanism by which cognition governs motor activity. Action planning is the intermediary process that connects ideation and motor execution, facilitating adaptive interactions with the environment (Ayres, 1961, 2011). Praxis involves the physical actions of engaging with the world and the conceptualization and planning of those motor activities (Bundy & Lane, 2020).

Occupational therapists evaluate praxis skills by observation, standardized scales, and tests (Bundy & Lane, 2020; Schaaf & Mailloux, 2015). The Sensory Integration and Praxis Test (SIPT; Ayres, 1989) and the Evaluation in Ayres Sensory Integration (EASI; Mailloux et al., 2018) are the main standardized tests occupational therapists perform to evaluate praxis in children. These assessments assess sensory perception and skills related to praxis, including imitation, sequencing, and following directions. Among these assessments, only the EASI possesses cultural adaptation and normative data for the Turkish population, indicating that Turkish occupational therapists have limited resources for evaluating praxis in children.

Consequently, additional praxis tests, particularly motor planning tests, are needed for use in the Turkish population. The Motor Planning Maze Assessment (MPMA; May-Benson, 2006) was created based on Ayres’ foundational research (Ayres, 1965) to fulfill the demand for a rapid, reliable, and cost-effective evaluation of motor planning skills (Ivey et al., 2014). Currently, this assessment is only available for use in English. There are data on American performance (Ivey et al., 2014; May-Benson et al., 2021) and a small sample from Canada (Buchner et al., 2024).

This study aimed to translate and culturally adapt the MPMA into Turkish in preparation for subsequent normative data collection.

## 2. Materials and Methods

A seven-step procedure for translation and cross-cultural adaptation informed by the literature review and recommendations (Beaton et al., 2000; Cruchinho et al., 2024; Peters & Passchier, 2006; Schuster et al., 2010; Swaine-Verdier et al., 2004) was developed. The procedure comprised forward and backward translations, professional linguistic analysis, cognitive interviews, and pilot testing. The study’s ethics committee received approval from the Clinical Research Ethics Committee (61351342/020-362) of Üsküdar University. Informed consent was acquired from all caregivers participating in the study.

### 2.1. Participants

A total of 24 unique individuals participated in the study: two forward translators, two backward translators, twelve reviewers, two testers, and ten children (see Table 1 for participant information).

### 2.2. Procedure

The seven-step process for translating and culturally adapting the MPMA was completed and is described below.

**Step 1:** The English MPMA was forward translated by two native Turkish speakers with excellent English language skills (Group 1, see Table 1 for details) who were aware of the study objectives. Both translators independently translated the original English version of the test sheet and instruction forms into Turkish.

**Step 2:** Both translations were synthesized and checked for comprehensiveness by Group 2. Discussion took place until a consensus was reached among all group members regarding the forward translation.

**Step 3:** The Turkish translations were assessed for grammatical accuracy and comprehensibility by a linguist and a special educator (Group 3). These translations were sent to the Step 2 Group who reviewed the edits and merged feedback into one document.

**Step 4:** The merged Turkish version was backward translated into English by two translators with extensive knowledge in the field (Group 4) and submitted to a team of reviewers (Group 5).

**Step 5:** Linguistic and semantic compatibility between back translations and the original version was assessed (Group 5). Terms and expressions that were ambiguous in relation to the original English version were identified, suggestions for clarification were made and, finally, the back-translation team (Group 4) reviewed and accepted the final Turkish version to produce the backward translated English version of the MPMA.

**Step 6:** All MPMA documents were checked for potentially necessary corrections or adaptations by experienced pediatric occupational and physical therapists (Group 6). Group 6 was asked to rate the understandability of scoring guidelines for the three test items included in the form on a 5-point Likert scale that ranged from 0 (very easy to understand) to 5 (very difficult to understand). The therapists also participated in cognitive interviews with group 5 regarding the material’s relevancy and comprehensibility for the Turkish population. Subsequently, they suggested phrases or words that would potentially enhance comprehension of the content.

**Step 7:** Cognitive interviews concerning the Turkish version were carried out by Group 7 with ten typically developing children aged between 6 and 8 years (Group 8) to determine if children correctly understood the instructions of the MPMA and consequently, the feasibility and applicability of using the MPMA in Turkish children. Children (Group 8) were presented with four questions with five-point Likert-scale responses (Strongly Agree, Agree, Neither Agree nor Disagree, Disagree, Strongly Disagree) regarding the comprehensibility of the test instructions, the test’s ease, its enjoyment aspect, and their willingness to retake the test.

### 2.3. Instruments

The MPMA was modified from wire grommet mazes initially created by A. Jean Ayres and utilized in her preliminary factor analyses of sensory integration (Ayres, 1965; Ivey et al., 2014; May-Benson, 2006). The MPMA is a valid and reliable individually administered assessment that evaluates the motor planning component of praxis in children aged from 3 to 12 years (Ivey et al., 2014; May-Benson et al., 2021). The evaluation is comprised of three mazes: a basic wire rectangle featuring a single handle (Maze 1—Rectangle), a complex twisted wire maze with a single handle (Maze 2—Single-Hand), and a twisted wire maze equipped with two handles (Maze 3—Two-Hand). Each maze features a small metal grommet on a wire structure, and the child is instructed to maneuver the maze to transfer the grommet from one side of the apparatus to the opposite side. The assessment requires roughly 5 min for administration and scoring. The scoring criteria are a combination of completion time and the errors exhibited. Each error exhibited is awarded one point, culminating in a maximum of four error points in each maze. Errors include shaking the maze, employing a hand to maneuver the grommet down the wire, transferring the maze to the opposite hand, and utilizing excessive bodily movements to maneuver the maze. Time scores are determined by allocating points based on completion time ranges, which differ by maze. The time scores and error values are combined to yield a total score for each maze. The maximum total score for Maze 1 is 10, for Maze 2 is 12, and for Maze 3 is 9, yielding a cumulative MPMA score range of 0–31 (May-Benson, 2006). Excellent inter-rater reliability has been demonstrated in the overall MPMA score (intraclass correlation coefficient = 0.96) and in individual maze scores (0.90–0.98) (Ivey et al., 2014).

## 3. Results

The results are presented in three sections, each corresponding to a phase in the translation process. The sections are as follows: forward translations, synthesis, grammatical precision, and comprehensiveness (Steps 1–3); back translation and a comparison between the back translation and the original test (Steps 4 and 5); and finally, cognitive interviews with therapists and children (Steps 6 and 7).

### 3.1. Forward Translations, Synthesis and Grammatical Precision

All members (100%) of the study’s synthesis group concurred that the two forward translations of the MPMA tests preserved significant conceptual closeness to the original English version. All test instructions were addressed to the therapists conducting the test. The synthesis team proposed minor word or term modifications or additions to nine out of twelve of the scoring criteria (75%) to enhance the clarity of the scoring criteria (see Table 2). In addition, the synthesis and linguistic expert group suggested integrating supplementary phrases to improve the clarity of some of the score items to improve therapists’ comprehension in rating and scoring items (see Table 3).

### 3.2. Backward Translation and Comparison with the Original English Version

The backward translation group, Group 5, in consensus with Group 6 determined that there were no semantic and conceptual differences between the backward translation and the original English version of the assessment. However, modifications to the scoring criteria that improved comprehension led to minor discrepancies in the exact wording of the descriptions of nine of the twelve instructions (75%) in the backward translation relative to the original form. A review with the test developer established that all minor wording modifications (100%) were acceptable (see Table 4).

### 3.3. Cognitive Interviews with Therapists

The cognitive interview was performed by Group 2 with five experienced occupational therapists and physiotherapists from Group 6 concerning the clarity of the test instructions. All therapists (100%) agreed that the directions were easy to comprehend. The two therapists who administered the Turkish version of the test to ten typical children aged from 6 to 12 years indicated that the test instructions were easy to comprehend and simple to implement. Both therapists (100%) noted that the children understood the test instructions effortlessly and considered the test enjoyable. Table 5 presents Group 8’s responses concerning understanding of the test instructions, the test’s ease, its enjoyment aspect, and their willingness to retake the test. All of the children in the group, except for one, responded to the questions with either “Strongly Agree” or “Agree”.

## 4. Discussion

This study provided a Turkish translation and cultural adaptation of the MPMA for use with Turkish children. The study followed a seven-step translation and cultural adaptation protocol, as outlined in the literature (Beaton et al., 2000; Cruchinho et al., 2024; Peters & Passchier, 2006; Schuster et al., 2010; Swaine-Verdier et al., 2004). All procedures were executed as suggested in the literature, yielding a Turkish version conceptually and semantically equivalent to the original English version of MPMA (May-Benson, 2006).

The MPMA is an effective motor planning assessment instrument for children, requiring minimal instruction for both the practitioner and the child (May-Benson, 2006). It significantly simplified the Turkish translation and cultural modification process, particularly in achieving conceptual and semantic equivalence.

The forward translation reviews indicated that the translations produced by both translators were consistent and adequately precise. The minimal instructions and simple scoring of the MPMA, along with one translator’s background as a therapist and the other’s extensive experience in translating for rehabilitation professionals, made the translation process very easy to complete (Papadakis et al., 2022). Nevertheless, the synthesis and linguistic analysis team implemented minor modifications, including several term replacements and additional explanations, to enhance clarity for therapists (Beaton et al., 2000; Cruchinho et al., 2024; Sousa & Rojjanasrirat, 2011).

Although there were no significant disparities in semantic and conceptual equivalence between the backward translation and the original English version of the MPMA, modifications made to the scoring items for improved comprehension resulted in discrepancies in the backward translation compared to the original version. Similar to our findings, modifications to the original text, even those aimed at improving comprehension, may unintentionally create linguistic and conceptual inconsistencies in the backward translation process (Sousa & Rojjanasrirat, 2011; Wild et al., 2019).

All therapists, including the review group and the administering therapist, concurred that the instructions were readily understandable. Furthermore, the therapist who conducted the assessment with children noted that administering it was uncomplicated. Additionally, almost all of the children in the group agreed that understanding the test instructions and doing the test was easy. They also stated that the test was enjoyable, and they wanted to retake it. Incorporating target experts and a target population during the cognitive interview phase of cultural adaptation guaranteed cultural relevance and accuracy while also facilitating the identification and adjustment of cultural biases, ambiguities, and misinterpretations that may compromise the integrity of the test (Hambleton & Zenisky, 2018; Maneesriwongul & Dixon, 2020).

Despite adhering to established principles for cultural adaptation, this study presents several limitations. First, the therapists who were interviewed and the therapist who administered the test to children were experienced in sensory integration and praxis concepts. This may limit understanding of the instructions by therapists who do not have training or experience in praxis. Furthermore, the fact that the test was administered only to typically developing children may have limited our ability to predict the responses of atypically developing children. Nevertheless, these participants sufficiently represent the populations that will use or be assessed by the MPMA instrument in Turkey. Finally, the seven-stage approach did not employ any generative AI technology. It may be intriguing to integrate AI technology with conventional methods in the cultural adaptation of questionaries or tests.

## 5. Conclusions

The findings of this study indicate that the Turkish version of the MPMA is an effective instrument for clinical practice and research in the Turkish population. The availability of suitably translated and culturally modified assessments, such as the MPMA, will impact practice and research prospects regarding motor planning in Turkey. Research that includes normative data, reliability, and validity for the Turkish community will improve the utilization of the MPMA among Turkish children.

## Figures and Tables

**Table 1:** Participants (groups), their respective steps, group size, participant characteristics, and assigned tasks.

Group	Step	n	Participants and Their Characteristics	Tasks
1	1	2	(1) An experienced bachelor’s level occupational therapist with over five years’ experience in pediatrics and with proficiency in English obtained by living in the UK for five years. (2) A medical translator, who graduated with a degree in English translation and interpretation with over 10 years of experience.	Forward translations of the test sheets and instruction forms. These participants only completed Step 1.
2	2	4	(1) The last author of the study, a physical therapist with a PhD, over 15 years of experience in pediatrics and a lecturer in the occupational therapy program at Fenerbahce university. (2) Two occupational therapists with master’s degrees and over 5 years of experience in pediatrics. One of them is the first author. (3) A physical therapist with a bachelor’s degree and over ten years of experience in pediatrics.	Synthesis of Turkish translations. Participants 1 and 2 (first author) completed Steps 2 and 5. The first author also engaged in step 7.
3	3	2	(1) A Turkish linguist with a degree in Turkish Language and Literature and over 20 years of experience teaching linguistics. (2) A special educator with a PhD in educational sciences and over 15 years of experience working with children.	Assessing the grammatical accuracy and comprehensibility of the Turkish translations. Participant 1 only completed Step 3 and Participant 2 completed Steps 3 and 5.
4	4	2	(1) A physical therapist with a bachelor’s degree and over 10 years of experience who serves as a professional continuing education course translator. (2) A master’s student in occupational therapy with over 5 years of experience in pediatrics and advanced knowledge of English obtained from living and working in the UK for a year.	Back translations of the Turkish versions. These participants only completed Step 4.
5	5	4	(1) The physical therapist (last author) from Step 2. (2) The occupational therapist (first author) from Step 2. (3) The special educator from Step 3. (4) The author of the MPMA, an occupational therapist with a PhD and 43 years’ experience in pediatrics and praxis and sensory integration.	Reviewing the linguistic and semantic compatibility between back translations and the original versions.
6	6	5	(1) Three bachelor’s level occupational therapists with certifications in sensory integration and praxis in pediatrics. (2) Two bachelor’s level physical therapists, one with over 10 years’ experience and the other with over five years’ experience. Both are certified in sensory integration and praxis with over 5 years’ experience in this specialty area.	Assessing for potential requisite modifications or adjustments. These participants only completed Step 6.
7	7	2	(1) The first author from Step 5. (2) Master’s student occupational therapist from Step 2. (3) Both were trained by the test author to administer the MPMA.	Testing children to determine practical feasibility and applicability and conducting cognitive interviews with children.
8	7	10	Children (4 females, 6 males) aged from 6 to 8 years, without any diagnoses, recruited from a private primary school in Istanbul.	Engaged in interviews and offered insights regarding their understanding of the translated materials.

**Table 2:** Words that were changed or added after forward translation.

Test or Test Item	Word or Term in Original English	Turkish Translation	Final Decided Turkish Translation
Level 1 Maze, Item 1	Grommet	Rondela	Halka
Level 2 Maze	Maze-Splat	Dalgalı Labirent	Dağınık Labirent

**Table 3:** Examples of scoring items that have been modified or enhanced with additional explanations.

Test or Test Item	Original English	Turkish Translation	Final Decided Turkish Translation
Level 1 Maze, Item 2	Child changes maze to other hand.	Çocuk, labirenti diğer eline geçirir.	Çocuk, labirenti diğer eline (terapistin labirenti yerleştirdiği elinden diğer eline) geçirir.
Level 1 Maze, Item 4	Child uses whole arm or body instead of wrist	Çocuk, bilek yerine kolunu ya da vücudunu kullanır.	Çocuk halkayı hareket ettirmek için sadece bileğini kullanmak yerine bütün kolunu ya da vücudunu kullanır.

**Table 4:** Examples comparing the back translation with the original English version.

Test Item	Original English	Turkish Final Translation	Decided Back Translation
Level 3 Maze, Item 2	The child removes the hand from the handle.	Çocuk bir elini tutamaktan çeker.	The child removes one hand from the handle.
Level 3 Maze, Item 3	Child uses other hand to move grommet.	Çocuk halkayı hareket ettirmek için bir elini kullanır.	The child uses one hand to move the ring.

**Table 5:** Distribution of Group 8’s answers to 4 questions.

Question	Strongly Agree	Agree	Neither Agree nor Disagree	Disagree	Strongly Disagree
1. I found the test instructions comprehensible. Children (n = 10)	9	0	1	0	0
2. This test was simple to complete. Children (n = 10)	9	0	1	0	0
3. I found the test enjoyable. Children (n = 10)	8	1	1	0	0
4. I wish to retake the test. Children (n = 10)	8	1	1	0	0

## Data Availability

All of the data are included in the manuscript.
